# *Leptodactylus validus* Garman, 1888 in Colombia: its distribution and identification

**DOI:** 10.3897/zookeys.737.20442

**Published:** 2018-02-13

**Authors:** Andrés R. Acosta Galvis, Rafael O. de Sá

**Affiliations:** 1 Subdirección de Investigaciones, Colecciones Biológicas,Instituto de Investigación de Recursos Biológicos Alexander von Humboldt, Claustro de San Agustín, Villa de Leyva, Boyacá, Colombia South América; 2 Department of Biology, University of Richmond, Richmond, VA 23173, USA

**Keywords:** Advertisement call, ecology, habitat, male nuptial spines, morphology

## Abstract

*Leptodactylus validus* is reported for the first time for Colombia, corresponding to the tenth species of the *L. melanonotus* species group occurring in the country. In collections, all *L. validus* specimens were identified as *L. colombiensis*. Morphological, coloration, and ecological characters are provided to differentiate the two species in Colombia. Furthermore, the distribution of *L. validus* is expanded based on the examination of specimens in both collections and literature records. In addition, the advertisement call of *L. validus* from Colombia is compared with those reported for other continental and insular populations; the calls are slightly more similar to those of insular populations.

## Introduction

Neotropical frogs of the genus *Leptodactylus* represent a clade consisting of 75 currently recognized species; 32 % of the species are known to occur in Colombia (de Sá et al. 2014). The genus was divided into four phenetic species groups (Heyer 1969). A recent and comprehensive phylogeny provided better understanding of species relationships and rearranged some species among groups to render each species group as monophyletic (de Sá et al. 2014). The *melanonotus* species group consists of 28 recognized species; nine of them have been reported for Colombia (de Sá et al. 2014).The *melanonotus* species group is recognized by five osteological cranial synapomorphies (de Sá et al. 2014). In Colombia, species of the *melanonotus* group are overall smaller (snout-vent length: 24.5–81.7 mm) than most species of *Leptodactylus* in Colombia. The examination of a large number of specimens from the “Llanos Orientales” of Colombia (i.e., extensive savannahs in northern South America extending between Colombia and Venezuela) showed the presence of *L. validus*. The species has not been reported for Colombia and, in collections, it is usually identified as *L. colombiensis*, Heyer 1994.

The presence of *Leptodactylus validus* in Colombia is reported and the characteristics that differentiate it from *L. colombiensis* are discussed. Information on the call, characteristics of the habitat, and distribution of *L. validus* in Colombia are provided.

## Materials and methods

Specimens herein identified as *L. validus* are deposited in the amphibian collections of the Universidad Javeriana, Bogotá (**MUJ**) and Instituto de Investigación de Recursos Biológicos Alexander von Humboldt, Villa de Leyva, (**IAvH-Am**); they were examined to understand the distribution of *L. validus* in Colombia. Measurements to 0.1 mm were taken with a digital Mitutoyo caliper. Measurements taken are:

**SVL** snout-vent length,

**HW** head width,

**HL** head length from tip of snout to the posterior border of skull (i.e., posterior edge of prootic, noted through the skin),

**IND** internarinal distance,

**IOD** interorbital distance,

**ED** eye diameter,

**END** eye-nostril distance,

**TD** tympanum diameter,

**UEW** upper eyelid width,

**ETS** distance between the anterior edge of the eye to the tip of snout,

**RW** rostral width,

**TL** tibia length,

**FL** femur length,

**FTL** foot length,

**HDL** hand length (measurements are provided as a Suppl. material 1).

Means are reported as +/- one standard error. Morphological terminology follows de Sá et al. (2014) and Heyer (1994). A small incision was made in the groin region to identify sex and sexual maturity through macroscopic observation of the gonads.

Two recordings were analyzed, both are deposited (i.e., BSA call vouchers) at the Colección de Sonidos Ambientales del Instituto Alexander von Humbdolt. Call voucher BSA-15988 corresponds to a specimen recorded on September 4, 2008, Municipio San Martin, Departamento de Meta, Colombia, 460 mts altitude (3°39.3'N, 73°36.51'W). The specimen was calling inside a water pond, although the recorded individual escaped collection temperature was recorded. Other vouchers individuals of the species were collected at the time of recording and are deposited with numbers MUJ6168-69 (Universidad Javeriana). Call voucher BSA-15994 corresponds to a specimen recorded and collected on June 4, 2012, Municipio Puerto Gaitan, Departamento de Meta, Colombia, 204 mts altitude (4°19.44’N, 71°43.54’W). The specimen was calling in a temporal pond, air temperate was 24.9°, and specimen voucher is deposited with number IAvH-Am-11347 (SVL = 38.4 mm). The terminology for call description follows Heyer (1994) and Yanek et al. (2006). Calls were recorded at constant levels with Marantz models PMD 671 and PMD 222 using Sennheiser models MKH 60 P40 and ME/80 directional microphones. The microphone was placed approximately one meter from the calling males. Air temperature and relative humidity were measured using a Data logger EXTECH Instrument RHT 10 graph with frequencies of recordings at 30 minute intervals. Digitization and editing were completed with a Windows digital signal analysis system at a sampling frequency of 44.1 kHz and 16 bits resolution; the analyses were made with Raven Pro 1.5 for Windows (Cornell Lab of Ornithology) using FFT (Fast Fourier Transformation) = 256 and Overlap = 50; coordinates taken with a Garmin GPS 60Csx. Distribution maps were constructed using ArcMap-Arcv.10.3.

## Results

**Figure 1.**
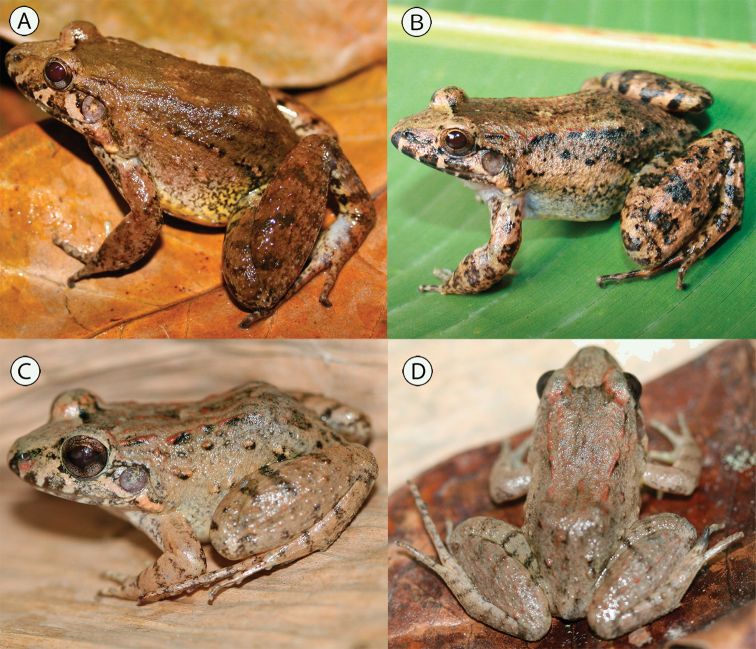
*Leptodactylus colombiensis*: **A** adult female MUJ 7225 **B** adult male, MUJ 7183. *Leptodactylus validus*: **C, D** adult female, IAvH-Am 11331.

**Figure 2.**
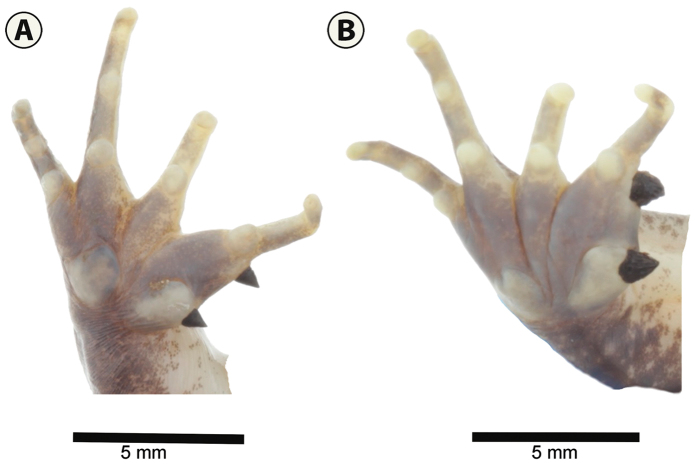
Ventral view of left hands showing nuptial spines in reproductive adult males. **A** *Leptodactylus validus*, IAvH-Am 11347 **B** *L. colombiensis*, IAvH-Am 8450. Scale bars: 5 mm.

*Leptodactylus validus* Garman, 1888 is a small species within the *L. melanonotus* species group; in Colombian populations adult females have a snout-vent length (SVL) of 25.3–49.5 mm (x̄ = 32.3 mm, N = 43) whereas in adult males SVL ranges between 25.0–40.8 mm (x̄ = 30.1 mm, N = 26); snout is short and rounded in dorsal view, not spatulate; distinctive light color stripes on the upper lip, including under the eye; males lack chest spines; a light color stripe extends ventrally from the corner of the eye and running anterior to the tympanum to the jaw articulation; this white band also extends over an elongated gland running between the jaw articulation and the point of attachment of the arm to the body; in addition a supratympanic gland, overall light brownish in coloration and, in some specimens, with a darker brown ventral edge, extends from the posterior edge of the eye, over the tympanum, and curving ventrally to the insertion of the arm; dorsal folds absent; dorsolateral folds usually short and interrupted, but rarely absent, in life they have a reddish to light coffee coloration; lateral folds absent; coloration of dorsum is overall a light brownish, posterior thighs and shanks dorsally with distinct and transverse dark bands (Fig. 1). During the breeding season, males exhibit a pair of small, black, pointed, smooth, and conical shaped thumb spines (Fig. 2A).

**Figure 3.**
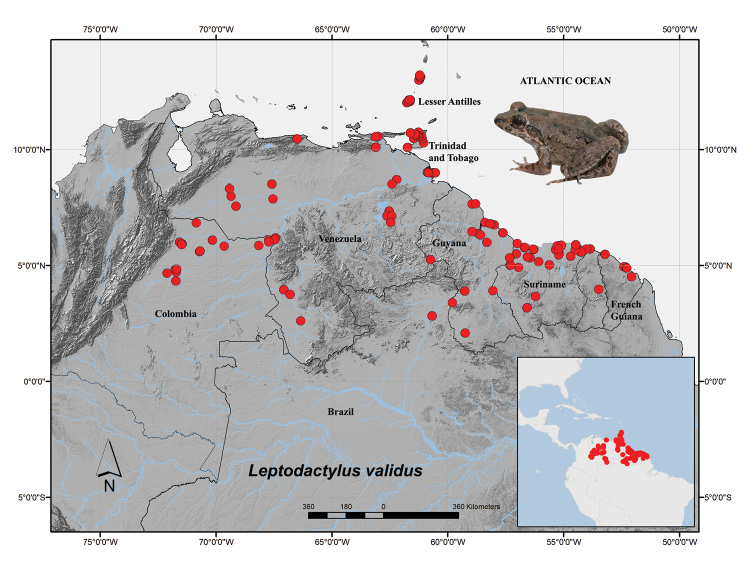
Distribution of *Leptodactylus validus*, Colombian localities in Suppl. material 1, other localities from Heyer (1994).

**Distribution in Colombia.** *Leptodactylus validus* occurs in north eastern South America; in Colombia the species is found in the western savannahs of Colombia, known as the “Llanos bajos” or “Llanos inundables” (Fig. 3). The western savannahs are an ecosystem consisting of alluvial and eolic flood plains that form the lowlands of the Orinoco basin. The species has been recorded in seven localities in the departments of Arauca, Casanare, Meta, and Vichada between 35–204 meters above sea level (Suppl. material 1).

**Figure 4.**
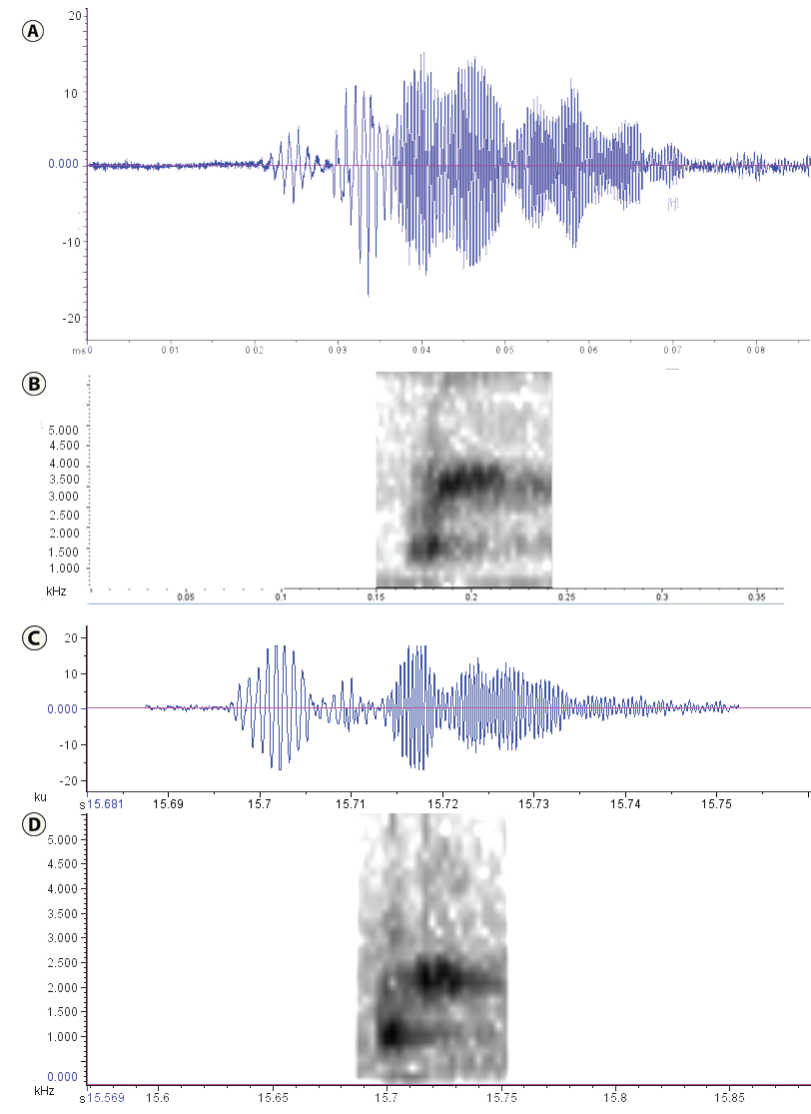
Advertisement calls: **A–B** *Leptodactylus validus*, BAS-15994 **C–D** *Leptodactylus colombiensis* (de Sá et al. 2014).

*Advertisement Call.* The advertisement call of *Leptodactylus validus* from populations in the western savannahs of Colombia consists of two notes. The first note consists of 1–3 pulses that are weak and increase gradually to the second note which consists of 2–6 pulses of wide amplitude that decrease gradually to lower amplitudes (Fig. 4A); the call rate is 1–3/s; the length of a call is between 0.03–0.069 s (x̄ = 0.046 ±0.008, N = 60 notes); time between calls ranges from 0.409 to 1.726 s (x̄ = 0.821 ±0.245, N = 48 notes); the dominant frequency ranges between 2392.2–3796.3 Hz (Fig. 4).

**Figure 5.**
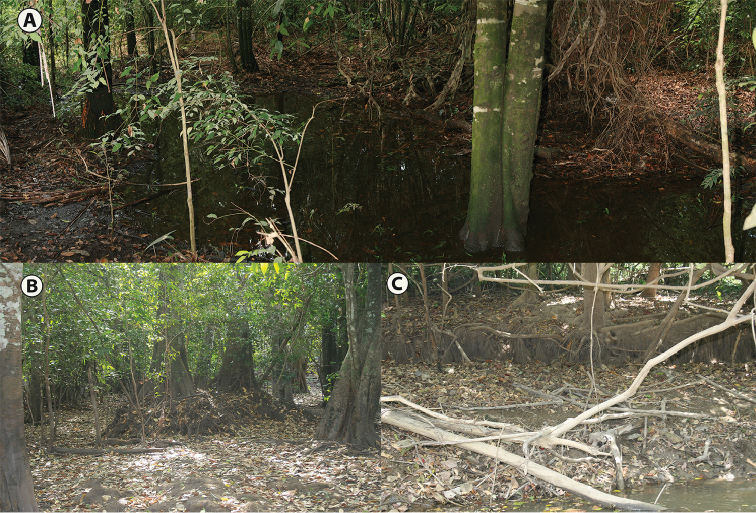
Habitats of *Leptodactylus validus* in the Oriental Llanos of Colombia. **A** Gallery forest during the rainy season, Puerto Gaitán, Meta **B** Gallery forest, during the dry season, Orocué, Casanare **C** Caño Canacavare, during the dry season, Orocué-Casanare.

*Habitat and ecology. Leptodactylus validus* occurs in transitional ecotones between gallery forests and: a) savannahs, b) “Morichales” floodplains (i.e., communities dominated by Moriche palms, *Mauritia flexuosa*), and c) eolic savannahs. During the dry season (December–March) the species was found actively moving at night among the leaf litter in flooded areas close to streams and creeks within gallery forests; but males were not calling. Throughout the rainy season (May–June), *L. validus* was found in large numbers and calling in flooded areas and temporal ponds within savannahs and ecotonal environments. Examination of specimens collected in the rainy season show that they were sexually mature, e.g., larger body size, males with prepollical spines, and females with large oviductal eggs. Specimens collected in the dry season have smaller body size and were collected among the leaf litter at the edges of streams or permanent ponds within gallery forests (Fig. 5); a large number of small size specimens collected in the dry season (December–March) were determined as juveniles through examination of internal sex organs.

## Discussion

Continental populations of *Leptodactylus validus* from Venezuela, Guyana, Surinam, and the north-central region of Roraima, Brazil, were identified as *L. pallidirostris* (Heyer, 1994). Furthermore, the author noted that a morphological similar species, *L. validus*, occurred in Trinidad and Tobago and across some of the oceanic islands of the Lesser Antilles (e.g., Sant Vicent, Bequia, Grenada). A study of *Leptodactylus pallidirostris* placed the species in the synonymy of *L. validus* (Yanek et al. 2006). It was proposed that *L. validus* spread from the mainland towards Trinidad (≈ 1 mya) followed by independent colonization events from Trinidad to Tobago and the Lesser Antilles (≈ 0.4 mya) mediated by human introductions (Camargo et al. 2009).

Acoustic parameters were described and compared for South American mainland populations (i.e., Brazil and Venezuela) and from the continental islands of the Lesser Antilles (i.e., Trinidad and Tobago) (Heyer 1994; Yanek et al. 2006). The calls have small differences when comparing continental vs. insular populations, e.g., broadcast frequency range: 1500–3500 Hz vs. 1300–3500 Hz; dominant frequency: 2500–3500 vs. 2300–3500; call duration: 0.03–0.05 vs. 0.03–0.06; and a second note consisting of 2 to 5 vs. 2 to 6 pulses (Heyer, 1994). Overall comparing with previously reported calls for the species (Heyer, 1994; Yanek et al. 2006), the calls of populations of *L. validus* from Colombia are slightly more similar to other continental populations in broadcast frequency range and call rate, whereas in other call parameters they are more similar to calls reported for insular populations.

Comparison of *L. validus* with *L. colombiensis*. Examinations found that in collections all specimens of *L. validus* had been misidentified as *L. colombiensis* based on external morphology. Interestingly, in the species accounts of the genus *Leptodactylus* (de Sá et al. 2014), the authors did not include either of these species in their respective sections of “similar species” which suggest that the species would not be easily confused. They characterized *L. validus* as a small to moderate size species (SVL: females x̄ = 38.4 mm, males x̄ = 35.2 mm) and *L. colombiensis* as a moderate size species (SVL female x̄ = 53.3 mm, male x̄ = 44.4 mm). Furthermore, molecular analyses recovered a clade of three genetically distinct taxa: *Leptodactylus validus*, *L. wagneri* and *L. colombiensis* (de Sá et al. 2014; Yanek et al. 2006) with *L. colombiensis* and *L. wagneri* as sister species.

**Figure 6.**
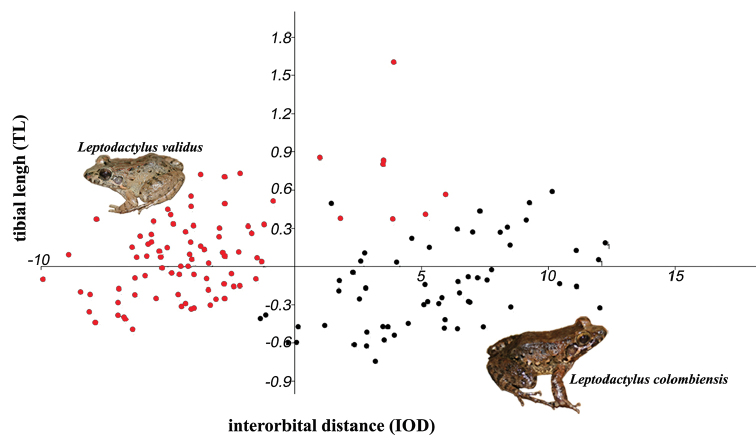
Separation of *Leptodactylus colombiensis* and *L. validus* (males and females) based on regression of interorbital distance (**IOD**) and tibial length (**TL**).

*Leptodactylus validus* in the Colombian savannahs can be distinguished from *L. colombiensis* by its smaller adult body size [*L. validus*: females SVL = 25.3–49.5 mm (x̄ = 32.3 mm, N = 43) and males SVL = 25.0–40.8 mm (x̄ = 30.1 mm, N = 26) and *L. colombiensis* females SVL = 39.88–60.44 mm (x̄ = 50.59 mm, N = 37) and males 34.1–52.9 mm (x̄ = 40.65 mm, N = 21)]. Furthermore, the interorbital distance (IOD)/length of the tibia (TL) ratio consistently separates the two species; this ratio was previously found useful to differentiate species of *Lithobates* (Hillis and de Sá, 1988). The IOD/LT ratio in *L. colombiensis* ranges between 13.6–17.7% (N = 58) whereas in *L. validus* is always greater (17.9–27.6%; N = 86; Fig. 6). In addition, the two species can be easily distinguished by their coloration in life, but preserved specimens are difficult to differentiate. Life coloration of *L. validus* is overall light brownish mostly lacking spots (Fig. 1) (darker brown with irregular shaped spots in *L. colombiensis*, Fig. 1), dorsal thigh coloration with darker and mostly distinct transverse bars in *L. validus* (wider and irregular in *L. colombiensis*, Fig. 1). *Leptodactylus validus* breeding males have smaller, conical, smooth, and pointed nuptial spines (Fig. 2A); whereas in *L. colombiensis* the nuptial spines are distinctly wider and non-smooth (Fig. 2B).

The advertisement calls of *L. validus* (as *L. validus* for insular populations and *L. pallidirostris* for mainland populations) have been previously analyzed (Heyer 1994; Yanek et al. 2006) and found to be very similar. The advertisement call of *L. colombiensis* was previously described (de Sá et al. 2014) and is similar that of *L. validus* since both have two notes consisting of up to eight pulses. In addition, the fundamental frequency in *L. validus* was reported to range between 1,500–3,500 Hz (Heyer 1994). The call of *L. colombiensis* has a smaller range between 1,470–1,980 Hz. Our analysis of *L. validus* calls from Colombia showed a fundamental frequency between 2,392 and 3,796 Hz (Fig. 4) and falls within the range of described calls for the species (Heyer 1994, de Sá et al. 2014). The call duration is 0.031–0.069 s and the calling rate of 1–3/s in *L. validus* (Fig. 4) whereas for *L. colombiensis* the reported call duration is 0.031–0.034 s and the call rate is 0.6 calls/s (de Sá et al. 2014).

**Figure 7.**
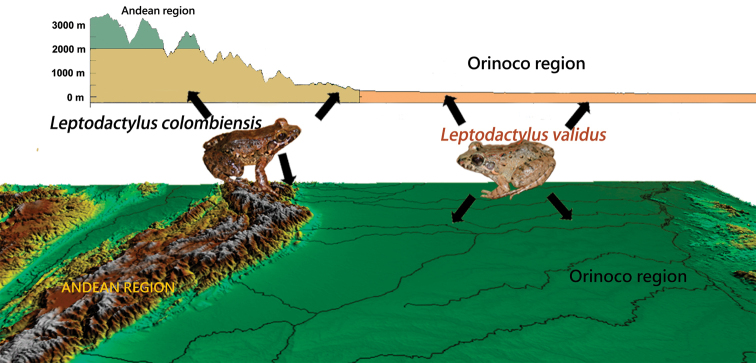
Eco-geographic distribution of *Leptodactylus colombiensis* and *L. validus* in Colombia.

Interestingly, distinct differences were found between the ecology and distribution of *L. validus* and *L. colombiensis* in Colombia. *Leptodactylus validus* has a strictly Cis-Andean distribution associated with the floodplains of the “Llanos Orientales”, that is savannahs with an altitudinal range from 35 m to 204 m and a precipitation of less than 2,800 mm (Fig. 7). Whereas the distribution of *L. colombiensis* is associated to Andean Mountains, 300 to 2,300 mts above sea level, with annual rainfall between 2,800–4,000 mm. Furthermore, *L. colombiensis* occupy piedmont (= “piedemonte”) natural forests and “rastrojos” (= scrub vegetation) in the eastern mountain range bordering with the “Llanos” floodplains corresponding to transitional environments toward “altillanuras”, i.e., Andean’s valleys and lower Caribbean mountain ranges.

The records for *Leptodactylus validus* reported here correspond to the western boundary of the species distribution (Fig. 3). The western savannahs of Colombia (“Llanos Orientales”) are geographically continuous with the Venezuelan’s savannahs. Overall, these floodplains are classified as “Llanos Savannahs” by WWF (Olson and Dinerstein 2002). All current localities for *L. validus* in the Colombian’s savannahs correspond to areas of lower altitude within the flooding ‘Llanos’ that are gradually continuous with the Savannas of the Guianan shield of Venezuela and Brazil. Until now, the understanding of the continental distribution of *L. validus* was restricted to the Guiana Shield (de Sá et al. 2014); however, our records of the species in several western localities in Colombia expand our understanding of the species distribution.

## Conclusions

*Leptodactylus validus* Garman, 1888 is reported to occur in the western savannahs of Colombia (= Llanos Orientales). The species has been commonly confused with *L. colombiensis* from which it can be distinguished by morphological, call, and ecological characteristics.

## Supplementary materials

Supplementary material 1 Measurements of *Leptodactylus validus* Data type: specimens data This is an open access article distributed under the terms of the Creative Commons Attribution License (CC BY 4.0), which permits unrestricted use, distribution, and reproduction in any medium, provided the original author and source are credited.Andrés R. Acosta Galvis, Rafael O. De Sá

## References

[B1] Camargo A de Sá RO Heyer WR (2009) Phylogeography of the frog *Leptodactylus validus* (Amphibia: Anura): patterns and timing of colonization events in the Lesser Antilles. Molecular Phylogenetics and Evolution 53: 571–579. https://doi.org/10.1016/j.ympev.2009.07.004 19596454 10.1016/j.ympev.2009.07.004

[B2] de Sá RO Grant T Camargo A Heyer WR Ponssa ML Stanley E (2014) Systematics of the Neotropical Genus *Leptodactylus* Fitzinger, 1826 (Anura: Leptodactylidae): Phylogeny, the Relevance of Non-Molecular Evidence, and Species Accounts. South American Journal of Herpetology 9(s1): S1–S100. https://doi.org/10.2994/SAJH-D-13-00022.1

[B3] Garman S (1888) “1887”. West Indian Batrachia in the Museum of Comparative Zoology. Bulletin of the Essex Institute 19: 13–16.

[B4] Heyer WR (1969) Studies on the genus *Leptodactylus* (Amphibia, Leptodactylidae). III. A redefinition of the genus *Leptodactylus* and a description of a new genus of leptodactylid frogs. Contributions in Science, Los Angeles County Museum of Natural History 155: 1–14.

[B5] Heyer WR (1994) Variation within the *Leptodactylus podicipinus-wagneri* complex of frogs (Amphibia: Leptodactylidae). Smithsonian Contributions to Zoology 546: 1–124. https://doi.org/10.5479/si.00810282.546.i

[B6] Hillis DM de Sá RO (1988) Phylogeny and taxonomy of the *Rana palmipes* group (Salientia: Ranidae). Herpetological Monographs 2: 1–26. https://doi.org/10.2307/1467024

[B7] Olson DM Dinerstein E (2002) The Global 200: Priority Ecoregions for Global Conservation. Annals of the Missouri Botanical Garden 89: 199–224. https://doi.org/10.2307/3298564

[B8] Yanek K Heyer WR de Sá RO (2006) Genetic resolution of the enigmatic Lesser Antillean distribution of the frog *Leptodactylus validus* (Anura, Leptodactylidae). South American Journal of Herpetology 1(3): 192–201. https://doi.org/10.2994/1808-9798(2006)1[192:GROTEL]2.0.CO;2

